# Molecular Imaging of Low-density Lipoprotein in Human Coronary Plaques by Color Fluorescent Angioscopy and Microscopy

**DOI:** 10.1371/journal.pone.0050678

**Published:** 2012-11-28

**Authors:** Yasumi Uchida, Yuko Maezawa, Yasuto Uchida, Nobuyuki Hiruta, Ei Shimoyama

**Affiliations:** 1 Japanese Foundation for Cardiovascular Research, Funabashi, Japan; 2 Research Institute, The Hospital for Sick Children, Toronto University, Toronto, Ontario, Canada; 3 Department of Cardiology, Toho University Ohmori Hospital, Otaku, Tokyo, Japan; 4 Department of Pathology, Toho University Sakura Hospital, Sakura, Japan; 5 Department of Pathology, Funabashi-Futawa Hospital, Funabashi, Japan; Brigham and Women’s Hospital, Harvard Medical School, United States of America

## Abstract

**Objectives:**

Low-density lipoprotein (LDL) is an important risk factor for coronary artery disease. However, its localization in human coronary plaques is not well understood. The present study was performed to visualize LDL in human coronary artery wall.

**Methods:**

(1) The fluorescence characteristic of LDL was investigated by color fluorescent microscopy (CFM) with excitation at 470-nm and emission at 515-nm using Nile blue dye (NB) as a biomarker. (2) Native LDL in 40 normal segments, 42 white plaques and 35 yellow plaques (20 with necrotic core) of human coronary arteries was investigated by color fluorescent angioscopy (CFA) and CFM.

**Results:**

(1) NB elicited a brown, golden and red fluorescence characteristic of LDL, apolipoprotein B-100, and lysophosphatidylcholine/triglyceride, respectively. (2) The % incidence of LDL in normal segments, white, and yellow plaques was 25, 38 and 14 by CFA and 42, 42 and 14 by CFM scan of their luminal surface, respectively, indicating lower incidence (p<0.05) of LDL in yellow plaques than white plaques, and no significant differences in detection sensitivity between CFA and CFM. By CFM transected surface scan, LDL deposited more frequently and more diffusely in white plaques and yellow plaques without necrotic core (NC) than normal segments and yellow plaques with NC. LDL was localized to fibrous cap in yellow plaques with NC. Co-deposition of LDL with other lipid components was observed frequently in white plaques and yellow plaques without NC.

**Conclusions:**

(1) Taken into consideration of the well-known process of coronary plaque growth, the results of the present study suggest that LDL begins to deposit before plaque formation; increasingly deposits with plaque growth, often co-depositing with other lipid components; and disappears after necrotic core formation. (2) CFA is feasible for visualization of LDL in human coronary artery wall.

## Introduction

Low-density lipoprotein (LDL) is a pro-atherogenic agent that is converted by oxidation to oxidized low-density lipoprotein (oxLDL), which plays an important role in the initiation, progression and destabilization of atherosclerotic plaques [1]–[4].

LDL is itself a clinically proven risk factor for ischemic vascular events; coronary heart disease is associated with higher serum levels of LDL [5], the incidence of silent myocardial ischemia is higher in type II diabetic patients with high levels of serum LDL [6]; and LDL is a predictor of future cardiovascular events in statin-treated patients [7]. However, all these data have been obtained by measuring the serum levels of LDL, not that which is deposited in the coronary arterial wall, and treatment has been directed to lowering the plasma levels of this substance in anticipation of reducing its content in the coronary arterial wall. If LDL could be visualized in the coronary arterial wall, its role in the initiation, progression and destabilization of atherosclerotic coronary plaques that cause coronary events could be clarified more objectively and the effects on plaque of medical and interventional therapies could also be evaluated more definitively.

Labeled-LDL has been used for trafficking LDL movement in fish, animal and humans [8]–[14], but molecular imaging of native LDL in the human coronary artery has never been carried out.

Although invasive, color fluorescent angioscopy (CFA) is a high-resolution imaging technique that enables percutaneous two-dimensional imaging of the vascular wall from within. We have succeeded in imaging the native lipid components, such as oxLDL, and lysophosphatidylcholine (LPC), of human coronary plaques by using CFA in vitro and/or in vivo, but have not attempted imaging of LDL because of the lack of an appropriate, biocompatible marker [15]–[18].

However, we recently discovered that Nile blue dye (NB), which is used as an electromechanical biosensor of DNA [19], for measurement of food antioxidant capacity [20] and for apoptotic cell staining [21], evokes a brown fluorescence when fluorescence is excited at 420±20-nm and emitted at 515-nm light wavelength. Therefore, in the present study, molecular imaging of native LDL in excised human coronary plaques by CFA was attempted using NB as a biomarker in anticipation of its clinical application. Color fluorescent microscopic (CFM) scanning of the luminal and transected surfaces of the same coronary plaques was also performed to clarify the deposition sites and patterns of LDL and their relation to plaque morphology studied by conventional angioscopy [22] As a result, deposition sites and patterns of LDL and their relation to plaque morphology were clarified considerably in human coronary artery wall by CFA and CFM.

## Methods

### 1. Investigating LDL by Color Fluorescent Microscopy

The color fluorescence of LDL and other major substances that constitute atherosclerotic plaques (Table 1) [23] was examined by means of CFM exciting at 470±20-nm and emitting at 515-nm using Nile blue dye (NB) as a biomarker. The details of CFM are described elsewhere [15], [16].

**Table 1:** Color Fluorescence of the Major Substances That Comprise Atherosclerotic Plaques after Addition of Nile Blue Dye (NB).

Substance			Autofluorescence	Fluorescence in thepresence of NB (10^−5^M)
Low-density lipprotein			no	Br
(lq; 20 mg/mL)				
Oxidized low-density lipoprotein			no	BLB
(lq; 2 mg/mL)				
Very low-density lipoprotein			no	no
(lq; 1 mg/mL)				
High-density lipoprotein			no	no
(lq; 11 mg/mL)				
Apolipoprotein B-100			no	Go
(lq; 1mg/mL)				
Apolipoprotein A-1			no	no
(lq; 2 mg/mL)				
Apolipoprotein E-2			no	R
(lq; 0.1 mg/mL)				
Phosphatidylcholine (p)			no	no
Lysophosphatidylcholine (p)			no	R
Triglyceride (p)			no	R
Cholesrerol (c )			W-to-LY	W-to-LY
Cholesteryl oleate (lq)			no	no
Cholesteryl linoleate (lq)			no	no
7-Keto-cholesterol (p)			no	no
Metalloproteinase-1, -9 (lq; 9 mg/mL)			no	no
Oleic acid (lq)			no	no
Linoleic acid (p)			no	no
Collagen I (f)			G	DG
Collagen III (f)			no	no
Collagen IV (f)			LG	LG
Collagen V (f)			no	no
Heparan sulfate (p)			no	no
Hyaluronic acid (p)			no	no
Albumin (p)			no	no
Globulins (p)			no	no
Ceramide (c)			Y	O
Calcium phosphate (c)			W-to-LY	W-to-LY
Hydroxyapatite (c)			no	O-to-Y
Elastin (c)			Y	no
β-Carotene			O	no

(c): crystal. (f): fiber. (lq): liquid. (p): powder.

B: blue. BLB: black-brown which masks other fluorescence including background fluorescence. Br: brown. DG: dark green. G: green. Go: golden. LY: light yellow. O: orange. R: red. W: white. Y: yellow. Bold characters: strong fluorescence. Non-bold characters: weak fluorescence.

no = no fluorescence.

Nile blue dye (NB) 10^−5^ M was added to each substance to evoke color fluorescence. Brown fluorescence was evoked by adding NB to low-density lipoprotein, but was not evoked by any of the other known substances that comprise atherosclerotic lesions. Golden fluorescence was exhibited by apolipoprotein B-100 alone. Red fluorescence was exhibited by lysophosphatidylcholine, triglycerides and apolipoprotein E-2, and they were collectively called “R” in the present study.

The intensity of fluorescence was categorized as strong, weak or absent when the exposure time required for imaging was taken to be within 1 s, >1 and 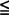
5 s, and >5 s, respectively.

In the present study, NB (Wako Co., Osaka, Japan) was diluted in distilled water to a concentration of 10^−5^ M (the maximum concentration that does not precipitate) at 37°C and then mixed with each of the major substances in atherosclerotic plaques. The evoked fluorescence was photographed at ×40.

### 2. Investigating LDL by Color Fluorescent Angioscopy

The CFA system we used consists of a fluorescence-excitation unit, an angioscope, a fluorescence-emission unit and a camera.^15^ The fluorescence-excitation unit (developed in collaboration with Olympus Co., Tokyo, Japan) comprises a mercury-xenon lamp and 7 sets of bandpass filter (BPF) discs, exchangeable by rotation to select the desired wavelength of light ranging from 330 to 800 nm.

The angioscope (modified VecMover, Clinical Supply Co., Gifu, Japan) consists of a 2.5-F fiberscope that contains 6000 quartz fibers for the image guide and 300 quartz fibers for the light guide. This fiberscope is incorporated in a 5-F guiding balloon catheter and is steerable along a 0.014-inch guide wire, which enables observation of a coronary segment of up to 7 cm in length with a single saline flush. The angioscope has been approved for clinical use by the Japanese Ministry of Health and Labor, supported by National Insurance, on a commercial basis in Japan [15].

The fluorescence-emission unit (developed in collaboration with Olympus Co.) comprises 7 sets of dichroic membranes (DM) and band absorption filters (BAF) and is connected to a 3CCD digital camera (C7780, Hamamatsu Photonics, Hamamatsu, Japan). The obtained images are displayed on a computer screen through a camera controller (C7780, Hamamatsu Photonics).

To observe the vascular lumen, the light and image guides are connected to the excitation and emission units, respectively. After selecting the desired BPF and BAF, the light is irradiated through the BPF and the light guide toward the target. The evoked fluorescence is received by the digital camera through the DM and BAF for successive two-dimensional imaging at an adequate time interval from 0.01 to 5 s. The intensity of the fluorescence images was categorized as for CFM.

This CFA system can visualize fluorescence of a target located within 200- µm of the luminal surface [16]. The limitation of the sensitivity of the CFA system was examined using the substances listed in Table 1 as the target, which revealed that their fluorescence was not detectable by the CFA system when their concentrations were 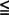
10^−6^ M [16].

### 3. Imaging of Coronary Plaques by Conventional Angioscopy

In the present study, conventional angioscopy was used to classify coronary plaques and normal segments because this imaging technology enables diagnosis of macroscopic pathological changes of the vascular wall from within, and therefore has been widely employed clinically for classification of coronary plaques and detection of vulnerable plaques in vivo [24]. The details of the system are described elsewhere [15].

Plaque is defined as a nonmobile, protuberant or lining mass that is clearly demarcated from the adjacent normal wall and the shape, location and color of which do not alter under the influence of a saline solution flush. Plaque is further classified as white or yellow based on its surface color. A normal segment is defined as milky-white and smooth-surfaced without any protrusions [22].

Images of the plaque obtained by conventional angioscopy were classified as white or yellow using an AquaCosmos image analyzer (C7746, Hamamatsu Photonics), which sets a window on an appropriate portion of an image and separates the color within the window into three primary colors, namely red, green and blue. Plaque was defined as “white” when the intensity ratio of red : green : blue was 1.0∶ 0.9∼1.1∶ 0.9∼1.1, respectively, and as “yellow” when it was 1.0∶ 0.8∼1.2∶ 0.3∼0.6, respectively [16].

### 4. Imaging of LDL in Excised Human Coronary Plaques by CFA

#### Ethics statement

The study was carried out with the approval of the Ethical Committees of the Japan Foundation for Cardiovascular Research, Ethical Committee of Toho University and Ethical Committee of Funabashi-Futawa Hospital. and after. obtaining written informed consent from the families concerned.

Twenty-nine coronary arteries (10 left anterior descending arteries, 9 left circumflex arteries, 10 right coronary arteries) were detached from 10 cadavers [61±3 years old, 3 females, 7 males; cause of death: acute myocardial infarction (2), aortic dissection (1), diabetic nephropathy (2), cerebral infarction (2), pancreatic carcinoma (1), hepatocellular carcinoma (2)].

A Y-connector was introduced into the proximal portion of each coronary artery for perfusion with saline solution at a rate of 10 mL/min and then the angioscope was introduced through the connector into the artery for observation. Initially, conventional angioscopy was carried out to detect plaque and because the light from the angioscope’s tip was visible through the coronary wall, the site of the targeted plaque could be confirmed.

As a result, 77 plaques (42 white plaques and 35 yellow plaques) and 40 normal segments were confirmed.

After conventional angioscopy, the CFA system was set up, a BPF of 470±20-nm and BAF of 515-nm were selected and a control image was obtained under saline perfusion. After ceasing the perfusion, 0.5 mL of 2% NB solution was injected into the perfusion circuit and 5 min later the perfusion of saline solution was restarted and the target portion was observed again.

Quantitative measurement of a target is difficult for CFA because the target was imaged as a fish eye image through a lens attached on the angioscope tip. Deposition pattern was therefore arbitrarily classified by CFA as spotty (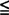 1/10 of a visual field), patchy (>1/10 and 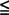
1/2 of a visual field) and diffuse (>1/2 of a visual field).

#### Definition of fluorescent color

Fluorescent color elicited by NB was analyzed by AquaCosmos image analyzer. Fluorescent color was defined as “brown” when the intensity ratio of red : green : blue was 1.0∶0.90∼1.25∶0.35∼0.50, respectively; as “gold” when it was 1.0∶ 0.85∼0.90∶ 0.15∼0.30 with glitter, respectively, and as “red” when it was 1.0∶ 0.09∼0.40∶ 0.09∼0.35, respectively.

### 5. Imaging of LDL by CFM

The 42 white plaques, 35 yellow plaques and 40 normal segments observed by CFA were isolated by transecting their proximal and distal ends along the shorter axes to avoid any damage to the plaque. The isolated segment was then cut longitudinally to open the lumen.

#### Scanning the luminal surface

The isolated coronary segments that contained plaques and normal segments were mounted on deck glass in such a way that the luminal surface faced the deck glass. The surface was then scanned by CFM to investigate the fluorescence of the substances listed in Table 1 at×40 or×10 using similar light wavelength filters as were used for CFA.

Quantitative measurement of the target can be performed by CFM luminal surface scan and its visual field at×40 is 850×600-µm^2^, deposition pattern of ApoB-100 was classified as spotty (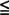
200-µm in diameter), patchy (>200-µm, 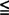
a half of the visual field) and diffuse (>half of visual field).

#### Scanning the transected surface

After observing the luminal surface by CFM, the center of each plaque was transected and one half was mounted on a deck glass in such a way that the transected surface faced the glass for CFM scanning to examine localization of LDL. Then, 0.1 mL NB solution was dripped onto the specimen. By this maneuver, the solution diffused to the transected surface and stained LDL.

Even in a normal human adult, the intima of the coronary artery is generally thickened. By CFM transected surface scan, the 40 normal segments were divided into 12 with thin intima (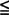
200-µm) and 28 thick intima (>200-µm). In plaques, the intima was divided into superficial (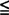 200 µm from luminal surface) and deep layer (>200-µm from luminal surface).The 35 yellow plaques were divided into 15 without necrotic core (NC) and 20 with NC.

In order to compare the images obtained by CFA, the site of deposition of LDL in the intima was classified as superficial (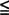
200-µm from the luminal surface), deep (>200-µm) or both, and the deposition pattern was termed superficial layer, deep layer or diffuse deposition, respectively.

The relationships between the deposition sites and patterns of LDL and plaque color determined by conventional angioscopy, intimal thickness and presence or absence of NC were examined.

### 6. Histology

After CFA and CFM scanning, each sample was cut into 10-µm slices along the shorter axis and stained with Oil Red O and methylene blue (MB), which stain lipids red, calcium black, and collagen fibers and smooth muscles blue [22], [24]. In a preliminary study, cholesterol, cholesteryl esters and ceramide stained red with Oil Red O, but not any of the other substances, including apolipoproteins and lipoproteins, listed in Table 1.

### 7. Statistical Analysis

The data obtained were tested by χ^2^ formulae and were compared among different deposition sites, patterns and plaque morphology. A value of p<0.05 was considered to be statistically significant.

## Results

### 1. Fluorescent Color of LDL with CFM

**Figure 1:**
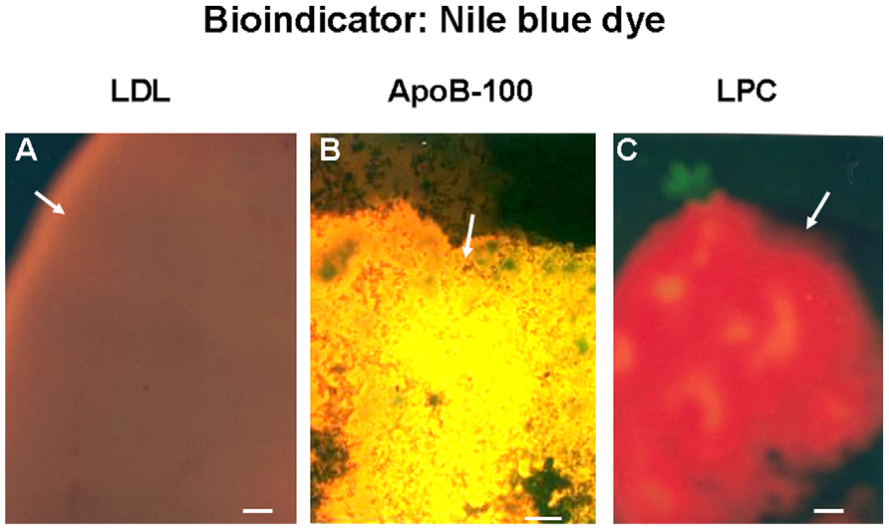
Fluorescent Colors of Low-density Lipoprotein (LDL), Apolipoprotein B-100 (Apo B-100) and Lysophosphatidylcholine (LPC). LDL, ApoB-100 and LPC did not exhibit autofluorescence, but in the presence of Nile blue dye (NB), LDL, ApoB-100 and LPC exhibited brown (arrow in A), golden (arrow in B) and red fluorescence (arrow in C), respectively. The brown fluorescence color of LDL and golden fluorescence color of ApoB-100 were not exhibited by any of the other known substances that comprise atherosclerotic plaques (listed in Table 1). Red fluorescence color was exhibited not only by LPC but also by triglycerides and apolipoprotein E-2. LPC, triglyceride and apolipoprotein E-2 are collectively termed as “R”. Bar = 100-µm.

LDL did not exhibit autofluorescence, but exhibited a brown fluorescence in the presence of NB (Fig. 1). This fluorescence color was not exhibited by any of the other major known substances that constitute atherosclerotic plaques (Table 1) [23], indicating that this color is characteristic only of LDL. Apolipoprotein B-100 (ApoB-100), an important constituent of LDL, had a golden fluorescence that was not presented by any of the other substances. LPC, triglycerides (TG) and apolipoprotein E-2 (ApoE-2) presented a red fluorescence. These substances which exhibit red fluorescence was termed as “R” (Fig. 1 and Table 1).

### 2. LDL in Excised Human Coronary Plaques Observed with CFA and CFM

#### Representative examples

**Figure 2:**
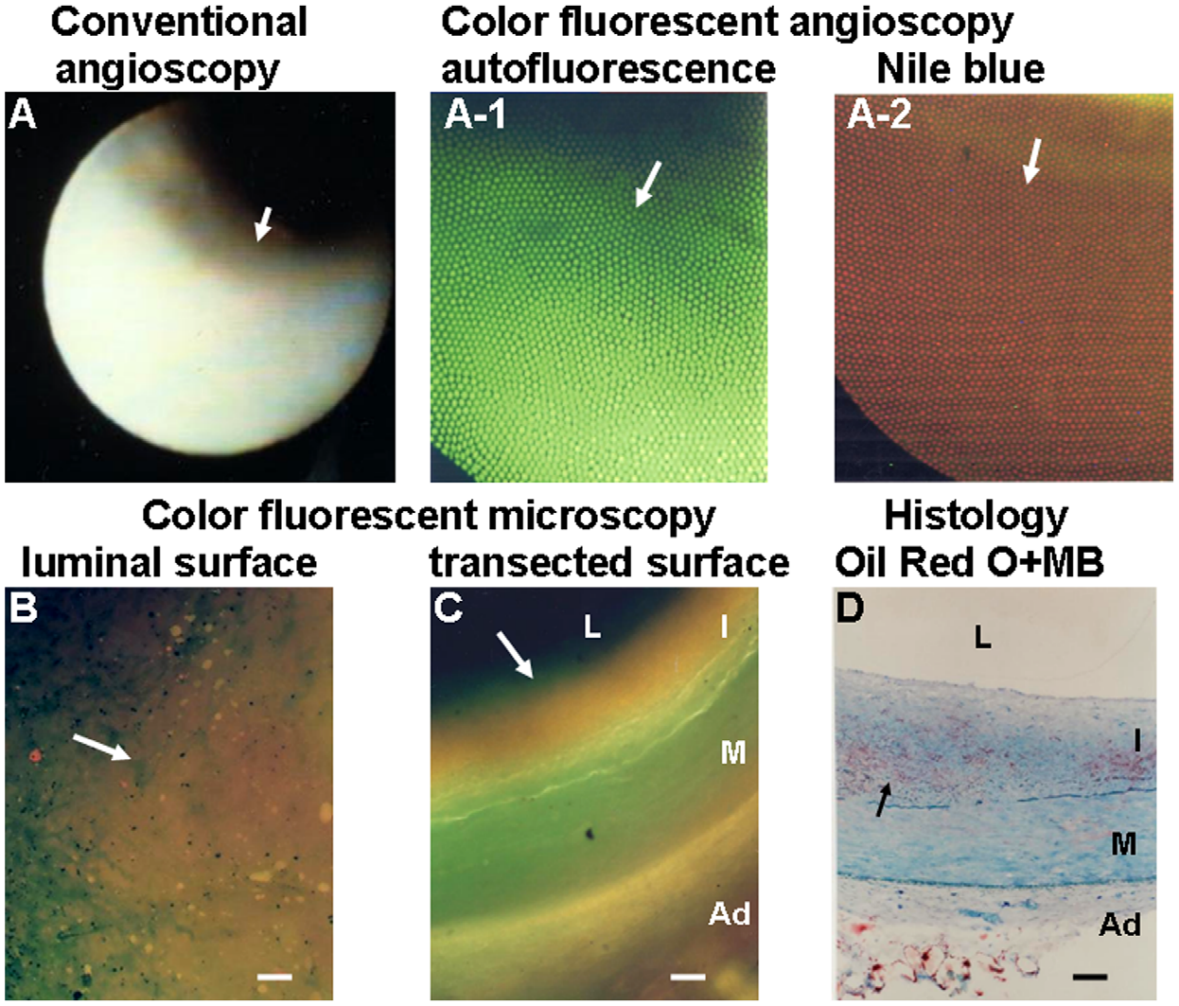
Low-density Lipoprotein (LDL) Visualized by Color Fluorescent Angioscopy (CFA) and Color Fluorescent Microscopy (CFM) in a Normal Coronary Segment. A: Normal coronary segment imaged by conventional angioscopy. Arrow shows the portion observed by CFA. A-1: CFA image of the same segment before administration of NB. The plaque shows green autofluorescence (arrow), indicating the existence of abundant collagen I and/or IV and the absence of lipids [15], [16]. A-2: CFA image after administration of NB. The segment shows diffuse brown fluorescence (arrow), indicating the existence of LDL. B: Scanning the luminal surface of the same segment by CFM shows diffuse deposition of LDL (arrow). C: Scanning the transected surface of the same segment shows diffuse deposition of LDL in intima (arrow). Histology of the same segment after Oil Red-O and methylene blue (MB) staining shows slight deposition of lipids in the deep layer (arrow) but not in the superficial layer of the intima where LDL is deposited, indicating that LDL deposits not only in lipid-deposited layers but also non-lipid-deposited layers. L, I, M and Ad indicate lumen, intima, media and adventitia, respectively. Bars = 100-µm.

**Figure 3:**
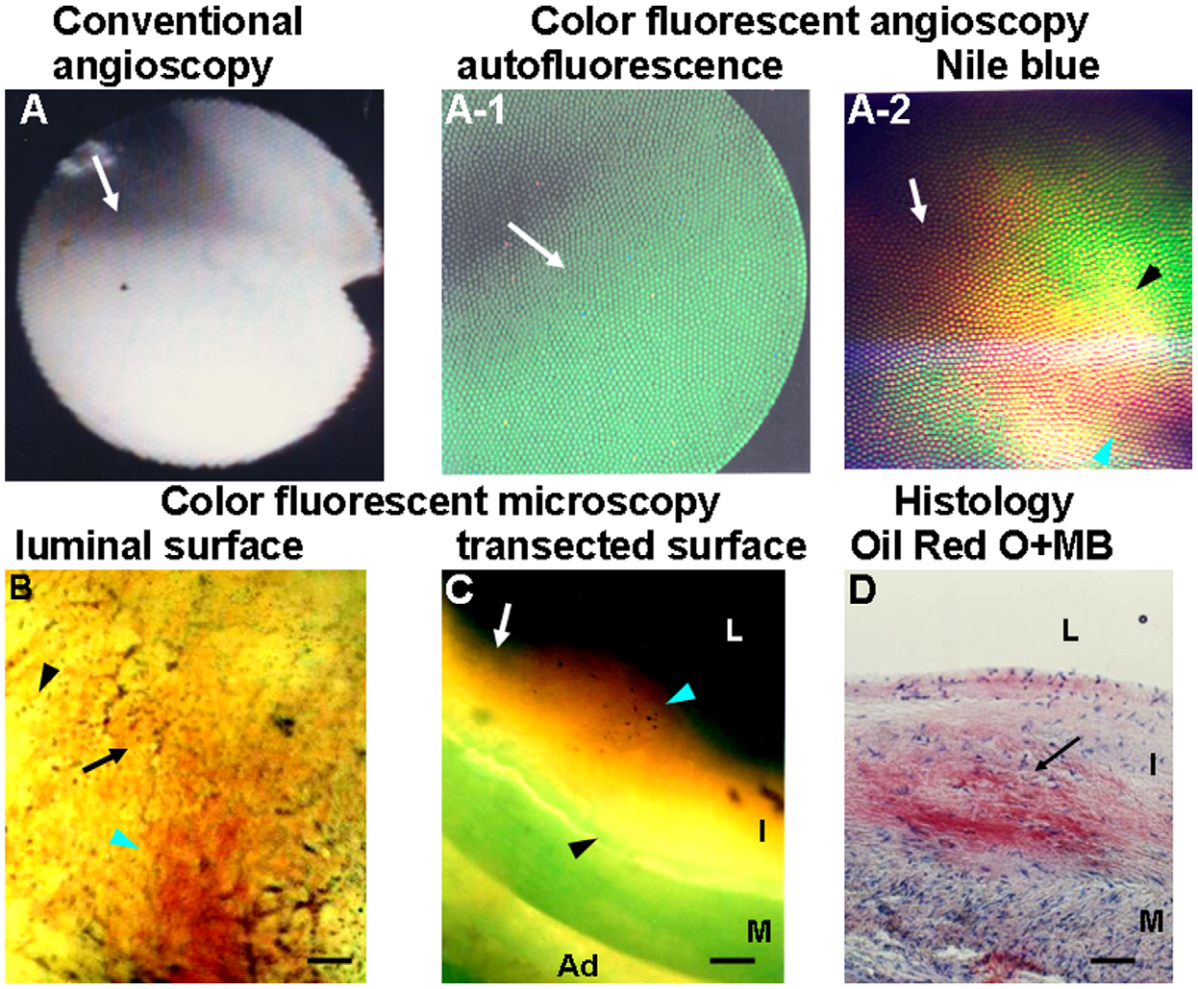
Co-deposition of Low-density Lipoprotein (LDL), Apolipoprotein B-100 (Apo B-100) and Other Lipid Components that Exhibit Red Fluorescence in White Coronary Plaque. A: White plaque imaged by conventional angioscopy (arrow). A-1: By CFA, the same plaque exhibits green autofluorescence, indicating the presence of abundant collagen I (arrow). A-2: CFA after administration of NB shows overlapping of brown (white arrow), golden (black arrowhead) and red fluorescence (green arrowhead), indicating co-deposition of LDL, and “R”. B: Scanning the luminal surface of the same plaque by CFM after NB administration also shows the co-deposition of LDL (arrow), ApoB-100 (black arrowhead) and “R”(green arrowhead). C: Scanning the transected surface of the same plaque after NB administration also shows the co-deposition of LDL (arrow), ApoB-100 (black arrowhead) and “R” (white arrowhead). D: Histology after staining by Oil Red O and MB shows mild deposition of lipids such as cholesterol and cholesteryl esters in the plaque (arrow). Bars = 100-µm.

Figure 2 is an example of the brown fluorescence indicating deposition of LDL alone as visualized by CFA and CFM scanning of the luminal and transected surfaces of a normal segment with an intimal thickness >200-µm. Figure 3 shows the co-existence of brown, golden and red fluorescent colors in a white plaque, indicating overlapping co-deposition of LDL, ApoB-100 and “R” (LPC, TG and ApoE-2).

#### The percentage incidence of LDL deposition

The % incidence of LDL deposition in normal segments, white plaques and yellow plaques were 25, 42 and 40 by CFA, 38, 42 and 42 by CFM luminal surface scan, and 14, 14, and 26 by CFM transected surface scan, respectively. The % incidence examined by CFM luminal and transected surface of normal segments was significantly higher that of CFA, and the reason for the difference was shown below. On the contrary, that in white plaques and yellow plaques was not different between CFA and CFM. By both CFA and CFM, the % incidence of LDL deposition in yellow plaques was lower than that of white plaques and normal segments (Table 2).

**Table 2:** LDL Visualized by Color Fluorescent Angioscopy (CFA) and Microscopy (CFM).

		Normalsegment		WhitePlaques		Yellow plaques
N		40		42		35
CFA		9		17†		6
(%)		(35)		(38)		(14)
Luminal surface		17*		18†		6
scan by CFM (%)		(42)		(42)		(14)
Transected surface		16†		8†		9
scan by CFM(%)		(40)		(42)		(25)

Normal segments, white plaques and yellow plaques were classified by conventional angioscopy using a white light as a light source. n = number of plaques or normal segments examined.

* p<0.05 vs CFA.

† p<0.05 vs yellow plaques.

#### Deposition patterns visualized by CFA and CFM luminal surface scan

**Figure 4:**
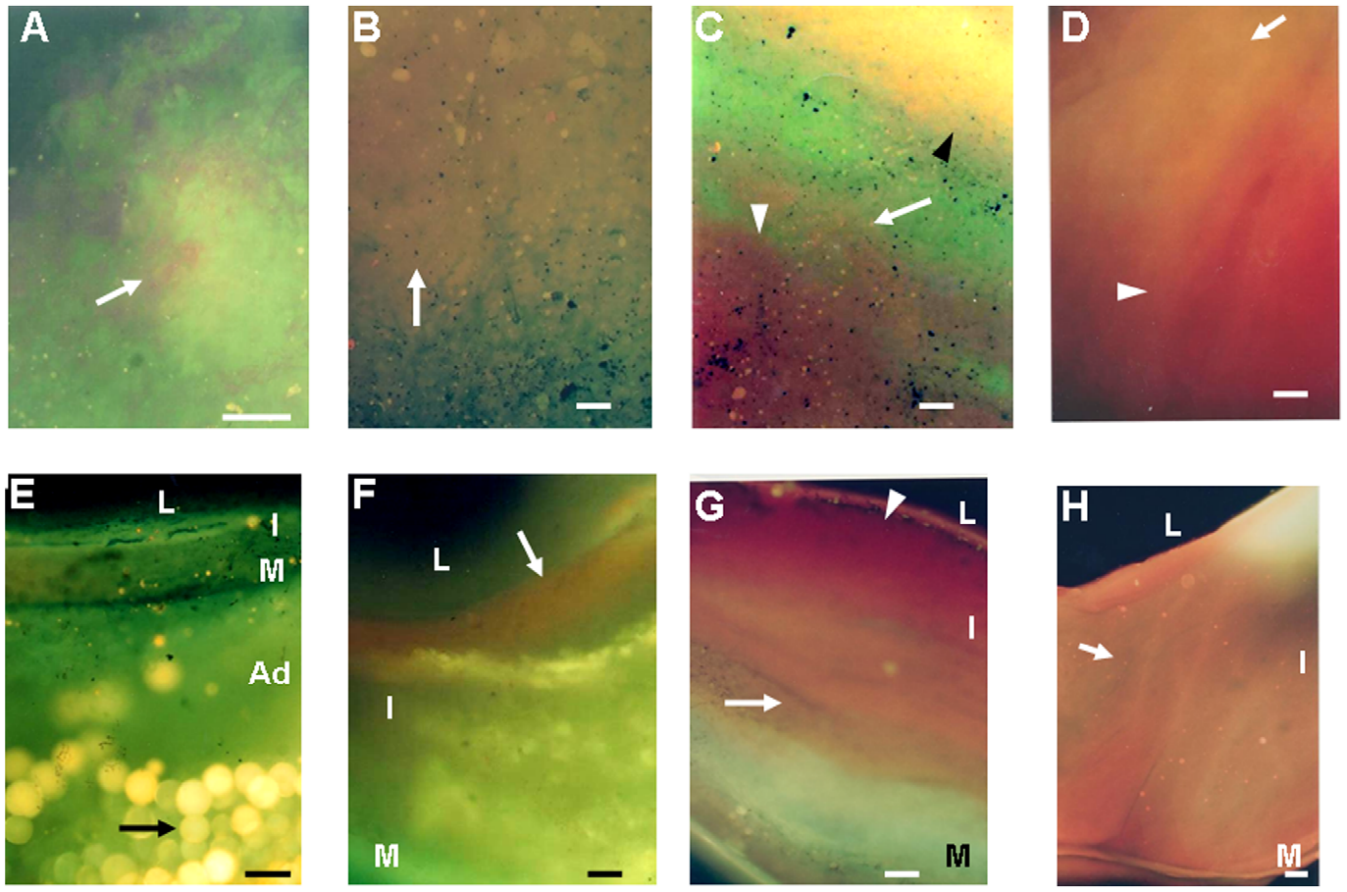
Deposition Patterns of Low-density Lipoprotein (LDL) in Coronary Arteries. Upper Column. Scanning of the luminal surface by CFM after administration of NB, showing (A) spotty type deposition of LDL (arrow), (B) diffuse type deposition of LDL (arrow), (C) separated co-deposition of LDL (arrow) with ApoB-100 (black arrowhead) and other lipid components that exhibited red fluorescence such as LPC, TG and ApoE-2 (white arrowhead), and (D) overlapping co-deposition of LDL (arrow) and “R” (arrowhead), respectively. Lower Column. Scanning of the transected surface by CFM after administration of NB showed (E) no deposition of LDL, but deposition of ApoB-100 in adipocytes (arrow), (F) deposition of LDL in the superficial layer of the intima (arrow), (G) deposition of LDL in the deep layer (arrow) with co-deposition of “R” in superficial layer (arrowhead), and (H) diffuse deposition of LDL in both layers (arrow). Bars = 100-µm.

By CFM luminal surface scan, LDL deposited alone or co-deposited separately or in overlapping pattern with ApoB-100 and “R” (Fig. 4A–D). Deposition pattern of LDL was classified as spotty, patchy and diffuse. Spotty deposition was observed in normal segments and white plaques but not in yellow plaques. The spotty deposition was not visualized by CFA. This was the cause of lower detection rate of LDL deposition by CFA.

Diffuse deposition was more frequent than spotty deposition in white plaques, and this deposition pattern alone was observed in yellow plaques (Table 3).

**Table 3:** Deposition Patterns of Low-density Lipoprotein (LDL) Visualized by Color Fluorescent Angioscopy (CFA) and Luminal Surface Scan by Color Fluorescent Microscopy (CFM).

		Normalsegments	WhitePlaques	Yellow plaques
Spotty deposition				
CFA (%)		0/9 (0)	0/17 (0)	0/6 (0)
CFM (%)		7/17 (41)* †	3/18 (15)	0/6 (0)
Patchy deposition				
CFA (%)		6/9 (66)	7/17 (41)	0/6 (0)
CFM (%)		6/17 (35)	5/18 (27)	0/0 (0)
Diffuse deposition				
CFA (%)		3/9 (33)	10/17 (63)	6/6 (100)
CFM (%)		4/17 (23)	10/18 (55)* ‡	6/6 (100)

* p<0.05 vs spotty type.

† p<0.05 vs yellow plaques.

‡ p<0.05 vs normal segments. Denominator: number of plaques or normal segments in which LDL was visualized.

#### Deposition sites of LDL visualized by transected surface scan by CFM

LDL deposited in superficial layer, deep layer or in diffusely in both layers, (Fig. 4-F–H), significantly more frequently in normal segments with thick intima and white plaques than in yellow plaques with NC, and also than normal segments with thin intima. Diffuse deposition was more frequent in white plaques than normal segments. The majority of LDL deposited in deep layer was not visualized by CFA. The LDL in superficial layer which was not visualized by CFA was spotty type as in case of CFM luminal surface scan (Table 4).

**Table 4:** Deposition Sites of Low-density Lipoprotein (LDL) Visualized by Transected Surface Scan by Color Fluorescent Microscopy (CFM).

Intimal thickness (µm)	Normal segments 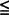 200	Normal segments>200	White plaques	Yellow plaquesNC(−)	Yellow plaquesNC(+)
Number of segments orplaques examined	12	28	42	15	20
LDL deposited (%)	2 (17)	14 (50)†	18 (42)†	6 (40)	3 (15)
Superficial layerdeposition (%)	2 (17)	8 (57)	5 (27)	1 (16)	2 (66)
Visualized by CFA	1	4	4	1	1
Deep layer deposition (%)	0 (0)	2 (14)	2 (11)	0 (0)	1 (33)
Visualized by CFA	0	0	1	0	0
Deposition in both layers (%)	0 (0)	4 (28)	11(61)*†	5 (83)	0 (0)
Visualized by CFA	0	4	11	4	0

* p<0.05 vs superficial layer deposition. †p<0.05 vs NC(+). NC(−): necrotic core absent. NC(+): necrotic core present.

#### Co-deposition of LDL with other lipid components

LDL deposited alone or with ApoB-100 in normal segments and white plaques but not in yellow plaques. In yellow plaques, LDL deposited with “R”, or with ApoB-100 and “R” (Fig. 4, and Tables 5 and 6). The fluorescent colors of LDL was diffusely distributed and did not Localize to endothelial cells, collagen fibers nor smooth muscle cells in normal segments and white plaques in which inflammatory cells were not observed (Fig. 2C and D). Round mononuclear cells, suggesting inflammatory cells, were distributed dot-like distribution (Fig. 3D), while the fluorescence of LDL, ApoB-100 and “R” distributed diffusely and did not localize to these cells (Fig.3B and C), indicating that these fluorescent colors were not elicited by the cells themselves.

**Table 5:** Co-deposition of Low-density Lipoprotein (LDL) with Other Lipid Components Visualized by Color Fluorescent Angioscopy (CFA) and Luminal Surface Scan by Color Fluorescent Microscopy (CFM).

		Normal segments		White plaques		Yellow plaques	
LDL alone (%)							
CFA		5/9 (55)		5/17 (29)		0/6 (0)	
CFM		9/17 (53)		6/18 (33)		0/6 (0)	
LDL withApoB-100 (%)							
CFA		2/9 (22)		4/17 (23)		0/6 (0)	
CFM		3/17 (17)		4/18 (22)		0/6 (0)	
LDL with “R” (%)							
CFA		1/9 (11)		5/17 (24)		5/5 (100)	
CFM		2/17 (12)		3/18 (17)		4/6 (66)	
LDL with poB-100and “R” (%)							
CFA		1/9 (11)		3/17 (17)		1/6 (16)	
CFM		3/9 (33)		5/18 (28)		2/6 (33)	

“R”: substances that exhibit red fluorescent color such as lysophosphatidylcholine, triglyceride and apolipoprotein E-2.

Denominator: number of plaques or normal segments in which LDL was visualized.

**Table 6:** Co-deposition of Low-density Lipoprotein (LDL) with Other Lipid Components Visualized by Transected Surface Scan by Color Fluorescent Microscopy (CFM).

		Normalsegments		Whiteplaques	Yellowplaques	
Intimal thickness		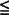 200	>200		NC (−)	NC (+)
(µm)						
N		2	14	18	6	3
LDL alone (%)		2 (100)	5 (36)	5 (28)	1 (16)	0 (0)
LDL withApoB-100 (%)		0 (0)	5 (36)	5 (28)	1 (16)	0 (0)
LDL with“R” (%)		0 (0)	1 (7)	2 (11)	1 (16)	2 (66)
LDL with ApoB-100 And"R" (%)		0 (0)	3 (21)	2 (11)	3 (50)	1 (33)

n = number of plaques or normal segmets in which LDL was visualized.

“R”: lipid components that exhibit red fluorescent color such as lisophosphatidylcholine, triglyceride and apolipoprotein E-2.

NC(−):necrotic cre absent. NC(+): necrotic core present.

#### Deposition of LDL with lipids stained by oil red o

Deposition of LDL without deposition of other lipids that are stained with Oil Red O, such as cholesterol and cholesteryl esters, was observed in normal segments, whereas in yellow plaques, the lipids deposited in all preparations but LDL did not necessarily co-localize with these lipid components (Table 7).

**Table 7:** Co-deposition of Low-density Lipoprotein (LDL) with Other Lipid Components That were Stained Red with Oil Red O and Methylene Blue Dye (MB).

		Normal segments	White plaues	Yellow plaques
N		40	42	35
Lipids (%)		4 (10)	20 (45)	35 (100)
LDL (%)		17 (42)*	19 (45)	10 (28)*

* p<0.05 vs Lipids. LDL deposition was examined by transected surface scan by CFM.

## Discussion

In the present study, NB, a low-molecular-weight dye, evoked a brown fluorescence in the presence of LDL. Thus, we were able to visualize LDL in a given plaque, enabling analysis of the different deposition patterns of LDL in human coronary plaques and their relationship to plaque morphology and deposition of other lipid components. The mechanisms by which NB evoked fluorescence by LDL are not known, but one possibility is that, as is the case of Evans blue dye with oxLDL [15], NB conjugates with LDL to form an adduct that provokes the brown fluorescent color.

Because none of the other major substances in plaque exhibited a brown fluorescence color, it is believed that it is characteristic of just LDL, and that NB can be used as a biomarker of LDL.

In this study, LDL was frequently deposited in normal segments with a thick intima, white plaques and yellow plaques without NC, more diffusely in white plaques and often co-depositing with ApoB-100 or other lipid components that exhibited red fluorescent color such as LPC, TG and/or ApoE-2 in white plaques and yellow plaques without NC and in normal segments with a thick intima, but infrequently in yellow plaques with NC.

LDL contains ApoB-100 in its cortex. In the present study, brown fluorescence of LDL and golden fluorescence of ApoB-100 distributed separately, indicating that golden fluorescence was not exhibited by the ApoB-100 that was contained in LDL but by free ApoB-100.

NB stains the damaged cells blue under sun light, irrespective of necrotic or apoptotic, as in case of Evans blue dye [25]. In a preliminary study, the damaged vascular endothelial cells and smooth muscle cells did not exhibit brown, golden nor red fluorescence in the presence of NB. In addition, the fluorescence colors observed in the present study did not localize to individual endothelial cells, collagen fibers and infiltrated mononuclear cells. Although fluorescence from the LDL, ApoB-100 and “R” that were incorporated in inflammatory cells cannot be denied, these evidences indicate that the fluorescence colors observed in the present study were not exhibited by the damaged cells themselves.

It is well known that white plaques are formed by intimal thickening caused mainly due to proliferation of collagen fibers; the white plaques grow into yellow plaques by accumulating lipids in themselves; acquire NC; and finally become vulnerable to disruption [22]. Therefore, the results in the present study indicate that LDL begins to deposit earlier than plaque formation, increasingly deposits singly or with other lipid components, and then its deposition decreases after NC formation. The reason for the decreased LDL in yellow plaques with NC is not known, but it is possible that it is converted to oxLDL by oxidation or is catalyzed [2]..Both white plaques and normal segments were classified as with or without LDL deposition and because no obvious differences were found by histology or the sex and age of the patients, it remains to be clarified whether the difference was attributable to other factors such as nutrition or underlying diseases.

Except LDL deposits with a small size and those located in deep layer, almost all LDL deposits that were visualized by luminal or transected surface scan by CFM were also detected by CFA in the present study. Although limited to significantly large sized (patchy or diffuse type) LDL deposits located in superficial layer, CFA is feasible for molecular imaging of native LDL in coronary artery wall in clinical situations.

The clinical safety of NB is unproven, unlike Evans blue dye, which is used as a biomarker of oxLDL [16], for staining fibrin [26], and as a safe and beneficial treatment of atherosclerosis [27]. Should NB become applicable clinically as a biomarker, the role of LDL in the initiation, progression and destabilization of coronary plaques and the effects of lipid-lowering drugs on the action of LDL can be clarified by CFA definitively.

### Conclusions

NB excited a brown fluorescence that was characteristic of LDL, which frequently deposited in white plaques and yellow plaques without NC and also in normal segment with thick intima, but rarely in yellow plaques with NC. (2) LDL often co-localized either separately or overlapping with ApoB-100, LPC, TG and/or ApoE-2. (3) Taken into consideration on the well known process of coronary plaque growth, it is conceivable that LDL begins to deposit before plaque formation, increasingly deposits with plaque growth, and then disappears after NC formation. (4) CFA is feasible for molecular imaging of native LDL deposits with a significantly large size and located in superficial layer of human coronary artery wall in vitro and in vivo.
